# Association of early PSA decline and time to PSA progression in abiraterone acetate-treated metastatic castration-resistant prostate cancer; a post-hoc analysis of Japanese phase 2 trials

**DOI:** 10.1186/s12894-016-0148-4

**Published:** 2016-06-08

**Authors:** Masahiko Nakayama, Hisanori Kobayashi, Tomihiro Takahara, Ryo Oyama, Keiichiro Imanaka, Kazutake Yoshizawa

**Affiliations:** Medical Affairs Division, Janssen Pharmaceutical K.K., 5-2, Nishi-kanda, 3-Chome, Chiyoda-ku, Tokyo, 101-0065 Japan; Research and Development Division, Janssen Pharmaceutical K.K., 5-2, Nishi-kanda, 3-Chome, Chiyoda-ku, Tokyo, 101-0065 Japan

**Keywords:** Abiraterone acetate, Castration-resistant prostate cancer, Kinetics, Prostate-specific antigen, Cox proportional hazard model

## Abstract

**Background:**

Previous studies have demonstrated an association between prostate-specific antigen (PSA) kinetics and predictive value for treatment outcomes. Abiraterone acetate (AA) is a newly approved cytochrome-P450C17 inhibitor for treatment of metastatic castration-resistant prostate cancer (mCRPC), and few studies have evaluated PSA kinetics using AA so far. Results of a study evaluating PSA kinetics in the beginning of AA and enzalutamide responded chemotherapy-treated patients suggested different trends between the drugs. PSA kinetics of AA-treated patients has been reported using large datasets; however, no studies which have fully evaluated PSA kinetics in the beginning treatment. The present study aimed to assess the PSA kinetics and relationship between the PSA kinetics and PSA progression in chemotherapy-naïve and chemotherapy-treated mCRPC patients receiving AA.

**Methods:**

We used two Japanese phase II trial datasets: JPN-201, chemotherapy-naïve mCRPC (*n* = 48) and JPN-202, chemotherapy-treated mCRPC (*n* = 46). PSA kinetic parameters were calculated using actual PSA values measured every 4 weeks, and a subgroup analysis was performed to evaluate the influence of early PSA response on time to PSA progression (TTPP). In addition, we used a Cox proportional hazard model to investigate the influence of variables on TTPP.

**Results:**

PSA declined from week 4 but took more time to achieve nadir. PSA kinetic parameters were different between the datasets, mean time to PSA nadir was 5.3 ± 5.6 and 2.0 ± 3.4 months, and TTPP was 9.5 ± 7.4 and 3.8 ± 4.8 months in JPN-201 and JPN-202, respectively. In the subgroup analysis of week 4 PSA decline status, Kaplan–Meier curves for TTPP were similar between early responders and non-progression patients in JPN-201 (median, 9.2 vs. 6.5 months, respectively) but separated in JPN-202 (median, 3.7 vs. 1.9 months, respectively). According to univariate Cox regression analysis, achievement of PSA response (≥50 %) at week 12 was associated with TTPP in the both trials, but the hazard ratio of PSA decline (≥30 %) at week 4 was not significant in JPN-201.

**Conclusions:**

Our results suggest that PSA kinetics were not comparable and early PSA response showed different association to TTPP according to prior history of chemotherapy.

**Trial registration:**

The original trials are registered at ClinicalTrials.gov. The identifiers are; JNJ-212082-JPN-201, registered 20 December 2012 and JNJ-212082-JPN-202, registered 30January 2013.

## Background

Globally, the estimated incidence of prostate cancer was approximately 1.4 million in 2013. There was a 3-fold increase in this incidence from 1990 to 2013, together with aging and population growth [1]. Since Huggins et al. discovered that prostate cancer growth is stimulated by androgens, castration has been the mainstay of advanced-stage prostate cancer treatment [2]; however, most patients develop resistance to castration.

Abiraterone acetate (AA) is a prodrug of abiraterone, which is a first-in-class inhibitor of cytochrome-P450C17 that plays a role in the mechanism of castration resistance by de novo androgen synthesis [3]. It is approved with prednisone for treatment of metastatic castration-resistant prostate cancer (mCRPC) worldwide. AA plus prednisone significantly prolonged overall survival (OS) compared with placebo plus prednisone for treatment of chemotherapy-naïve and chemotherapy-treated mCRPC in pivotal global trials [4, 5]. In Japan, two single-arm, open-label, phase II trials were separately conducted for the purpose of obtaining local registration [6, 7].

Prostate-specific antigen (PSA) is a reliable, sensitive, and easy to measure biomarker for prostate cancer and is therefore widely used for evaluation of treatment in practice [8, 9]. PSA kinetics has been studied in androgen deprivation therapy using anti-androgens or taxanes to analyze its predictive value for time-dependent outcomes such as OS and disease progression. Several studies have reported strength of PSA decline and its predictive value for OS, although certain results were controversial [9–11]. Recently, Caffo et al. reported the PSA kinetics of AA and enzalutamide responders and demonstrated different trends with regard to PSA kinetics between the drugs in chemotherapy-treated mCRPC patients [12]. However, patient number was limited, and PSA kinetics of chemotherapy-naïve mCRPC patients was not reported. Xu et al. also reported PSA kinetics of AA-treated mCRPC patients separately by chemotherapy-naïve and -treated populations. However, PSA kinetics within 12 weeks was not evaluated because the original trials measured PSA values every 12 or 16 weeks [13]. Thus, PSA kinetics in AA-treated mCRPC patients has not been fully clarified so far.

Moreover, the Prostate Cancer Clinical Trial Working Group (PCWG2) advises to ignore early PSA changes to avoid detecting continuing rise of PSA level and increasing in size before it regress [8]. However, some researchers reported early PSA decline, and its predictive value was possibly different by patient backgrounds and treatment [12, 14, 15]. In addition, the clinical practice in Japan, most of mCRPC patients are primary followed by monthly PSA testing, so, there is a potential of over use of early response as predictive factor for efficacy regardless the PCWG2 criteria.

The aims of the present study were to assess the PSA kinetic profile, and the relationship between PSA kinetics calculated based on actual PSA values measured 4 weeks and 12 weeks and PSA progression in chemotherapy-naïve and chemotherapy-treated mCRPC patients receiving AA.

## Methods

### Data source

This post-hoc study was conducted using datasets from two Japanese phase II trials of AA for mCRPC: JPN-201 (ClinicalTrials.gov Identifier, JNJ-212082-JPN-201) that included chemotherapy-naïve patients (*n* = 48) and JPN-202 (ClinicalTrials.gov Identifier, JNJ-212082-JPN-202) that included patients who received docetaxel-based chemotherapy (*n* = 46). Results from the original trials are available elsewhere [6, 7]. Major inclusion criteria were as follows: men with mCRPC aged ≥ 20 years with a PSA level of ≥ 5 ng/mL and an Eastern Cooperative Oncology Group Performance Status (ECOG-PS) score of 0 or 1 for JPN-201 and 0 to 2 for JPN-202, histologically or cytologically confirmed adenocarcinoma of the prostate without neuroendocrine differentiation or small cell histology, and testosterone levels of ≤ 50 ng/dL by medical or surgical castration. Eligible patients were orally administered AA 1000 mg with 10-mg prednisolone per day. For patients with medical castration, castration was maintained by using a luteinizing hormone-releasing hormone (LHRH) agonist throughout the study. PSA was assessed at baseline and every 28 days after the AA dose.

The primary endpoint of the original trials was the proportion of patients achieving a PSA decline of ≥ 50 % from baseline within 12 weeks of therapy in accordance with the Prostate-Specific Antigen Working Group criteria (PSAWG) [16].

An independent ethics committee or institutional review board approved the protocols of original trials and informed consent forms, and the trials were conducted in accordance with the ethical principles in the Declaration of Helsinki and consistent with the Good Clinical Practices and applicable regulatory requirements. All patients or their legally acceptable representatives provided written informed consent, which included consent for post-hoc analysis before entering the original trials.

### PSA values used in the study and definitions of PSA kinetic parameters

We used PSA values obtained after each 28-day cycle from baseline (day 0) to end of treatment (EOT) or study cutoff date (the longest duration was 785 days) to calculate the PSA kinetic parameters.

The following PSA kinetic parameters were calculated, as defined in the present study:

1. Maximum percentage PSA decline (%; defined as nadir PSA value/baseline PSA value × 100)
2. Time to PSA nadir (months; defined as duration from baseline to PSA nadir)
3. Nadir PSA value (ng/mL; defined as the minimum PSA value during the treatment period)
4. EOT PSA value (ng/mL; defined as the last PSA value at EOT)
5. PSA response (≥50 %) according to PCWG2 criteria
6. PSA response (≥30, 50, and 90 %) at week 12 according to PCWG2 criteria
7. Time to PSA progression (TTPP; months, defined as duration from baseline to the day of PSA progression according to PCWG2 criteria)

The purpose of the original studies was to demonstrate similarity with global trials, response criteria needed to be identical with global trials used. In the present study, we referred PCWG2 criteria instead of PSAWG criteria, because it no longer used in the current clinical practice.

### Statistical analysis

Findings from JPN-201 and JPN-202 were separately analyzed. Patient demographics, baseline characteristics, and PSA kinetic parameters were descriptively summarized using mean, standard deviation (SD), and percentage values. The percentage PSA change was longitudinally described based on mean (SD) and 95 % confidence intervals (CI) at each time point to characterize the post-treatment PSA kinetics.

We analyzed the impact of early PSA decline on TTPP. Because treatment was repeated every 28 days, and PSA was measured on the first day of each cycle in JPN-201 and JPN-202, the PSA value at week 4 was the earliest PSA value available to assess PSA transition. We subdivided the percent PSA change at week 4 into the following three subgroups: (1) PSA decline ≤ −30 % (30 %-decline), (2) PSA > −30 and < 25 % (non-decline), and (3) PSA elevation ≥ 25 % (25 %-elevation). Furthermore, TTPP was analyzed based on PSA response (≥50 %) at week 12 according to the PCWG2 criteria. In addition, impact of the PSA kinetic parameters on TTPP was investigated using a Cox proportional hazard model to obtain the hazard ratio (HR) and its 95 % CI. TTPP curves were developed using Kaplan–Meier method.

All statistical analyses were performed using R version 3.1.0 (a language and environment for statistical computing; R Foundation for Statistical Computing, Vienna, Austria).

## Results

### Baseline demographics and characteristics

In the present study, data from a total of 94 patients were analyzed: 48 patients from JPN-201 and 46 from JPN-202. Baseline demographics and characteristics are summarized in Table 1. In JPN-201, the median age was 70 years, 83.3 of the patients had ECOG-PS scores of 0, and 89.6 % had a Gleason score of ≥ 8. In JPN-202, the median age was 71 years, 52.2, 34.8, and 13.0 % of the patients had ECOG-PS scores of 0, 1, and 2, respectively, and 78.3 % had a Gleason score of ≥ 8.

**Table 1:** Baseline patient demographics and characteristics

	Chemotherapy-naive	Chemotherapy-treated
	JPN-201 (*n* = 48)	JPN-202 (*n* = 46)
Median age (range), years	70 (46–89)	71 (51–83)
Gleason score		
< 7	0 %	0 %
7	8.3 %	17.4 %
≥ 8	89.6 %	78.3 %
Unknown	2.1 %	4.3 %
ECOG-PS score		
0	83.3 %	52.2 %
1	16.7 %	34.8 %
2	–	13.0 %
Extent of disease		
Bone	91.7 %	95.7 %
Hepatic	2.1 %	4.3 %
Lymphatic	39.6 %	37.0 %
Pulmonary	0 %	10.9 %
Other	0 %	6.5 %
Median months from initiating LHRH agonist to first dose (range)	21.91 (6.2–191.6)	41.23 (4.4–182.8)
Median baseline PSA (range), ng/mL	31.40 (6.0–469.0)	147.00 (7.2–1450.0)
Median baseline hemoglobin (range), g/dL	12.85 (10.2–15.2)	11.80 (9.0–14.9)
Median LDH (range), IU/L	212.0 (164–1045)	211.0 (122–723)
Median alkaline phosphatase (range), IU/L	292.0 (139–2643)	327.0 (69–2991)
No. of previous chemotherapy regimens		
1	–	39.1 %
2	–	60.9 %

*ECOG*-*PS* Eastern Corporative Oncology Group Performance Status, *LDH* lactate dehydrogenase, *LHRH* luteinizing hormone-releasing hormone, *PSA* prostate-specific antigen

In JPN-202, all patients had received docetaxel-based chemotherapy at study entry.

### Abiraterone compliance, dose reducations and interaptions

In JPN-201, 91.7 % (44/48) had > 90 % compliance with AA. Dose reductions were required for 3 (6.3 %) patients. Dose interruptions were required for 21 (45.7 %) patients, once for 13 and 9 patients required 2 or more dose interruptions.

In JPN-202, 93.5 % (43/46) had > 90 % compliance with AA. Dose reductions were required for 6 (13.0 %) patients. Dose interruptions were required for 22 (47.8 %) patients, 8 patients required 2 or more dose interruptions.

No patients discontinued study treatment because of poor compliance in the both trials.

### PSA kinetic parameter values

PSA values were well followed up, PSA progression was confirmed 75 (36/48) and 10 % (5/48) was censored within the first year in JPN-201. In JPN-202, PSA progression was confirmed 93 (43/46) and 4 % (2/46) was censored within the first year.

**Fig. 1:**
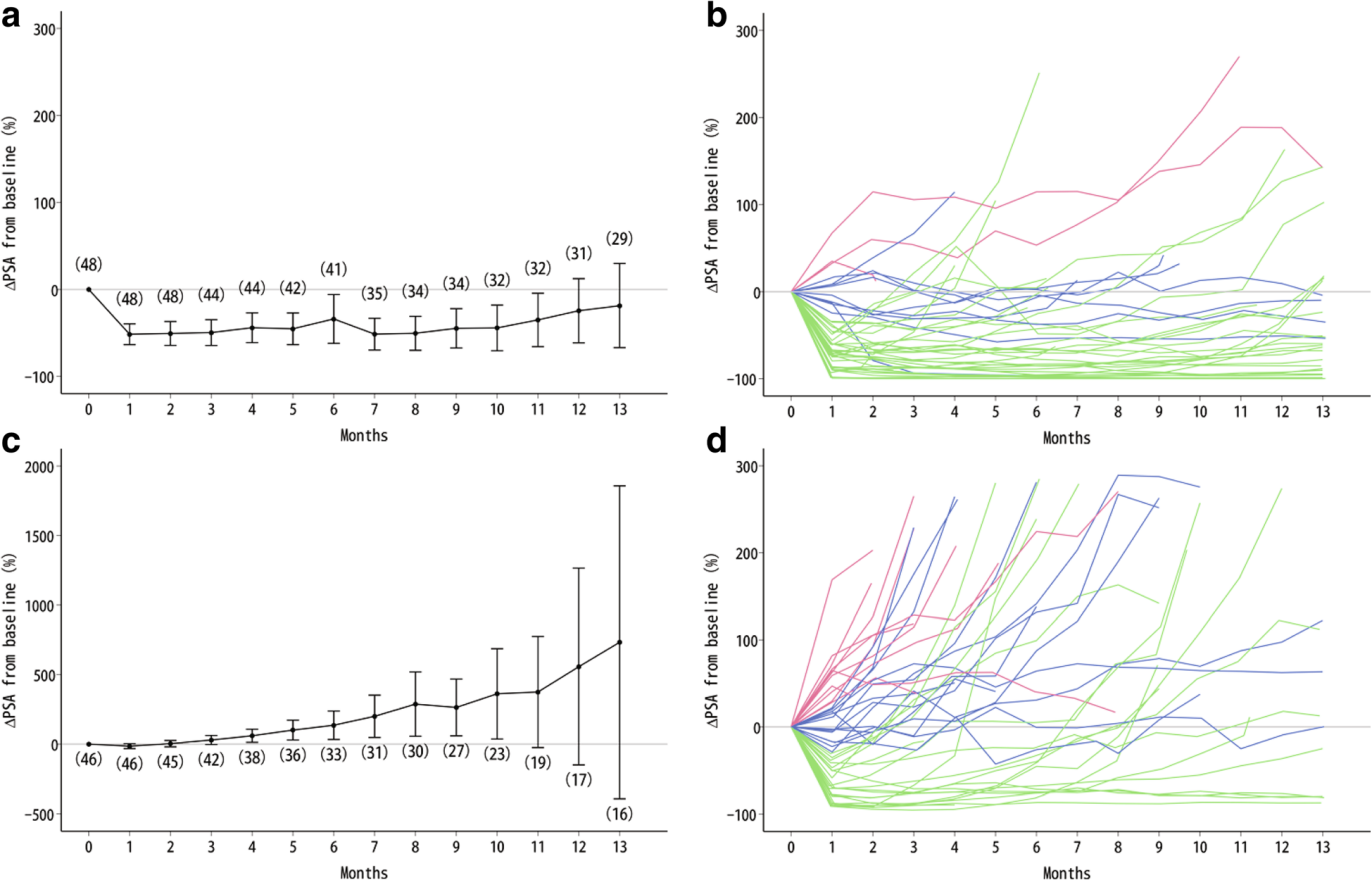
Percent PSA transition from baseline to month 13. **a** Mean percent PSA change in JPN-201, (n). **b** Percent PSA change for each patient in JPN-201; green, PSA ≤ −30 % (30 %-decline); blue, −30 % < PSA < 25 % (non-decline); red, PSA ≥ 25 % (25 %-elevation) according to PSA change at week 4. **c** Mean percent PSA change in JPN-202, (n). **d** Percent PSA change for each patient in JPN-202; green, PSA ≤ −30 % (30 %-decline); blue, −30 % < PSA < 25 % (non-decline); red, 25 % ≥ PSA (25 %-elevation) according to PSA change at week 4. Data are expressed as mean ± 95 % CI. Note: PSA changes of ≥ 300 % and those after month 13 are not shown

**Fig. 2:**
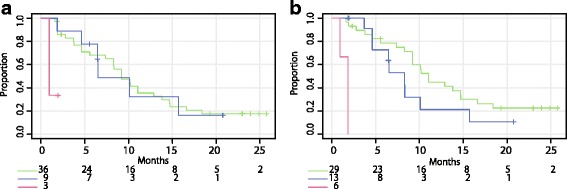
Kaplan–Meier curves for time to PSA progression in JPN-201. **a** green, PSA ≤ −30 % (30 %-decline); blue, −30 % < PSA < 25 % (non-decline); red, PSA ≥ 25 % (25 %-elevation) according to PSA change at week 4. **b** green, PSA response (≥50 %); blue, PSA non-response/progression; red, PSA progression according to PSA response at week 12

In JPN-201, PSA rapidly decreased from week 4 onwards, and the mean PSA decline from baseline value was 51.6 % (SD: 41.2) (Fig. 1a). Figure 1b depicts the percentage change of PSA transition in each patient. The PSA kinetic parameters are shown in Table 2. At week 4, 36 patients achieved a 30 %-decline, 9 patients showed a non-decline, and 3 patients showed a 25 %-elevation. PSA responses (≥50 %) were confirmed in 28 of 36 patients who achieved 30-decline, 2 patients who showed a non-decline, and none of the 25 %-elevation patients. Figure 2a shows the TTPP according to the week 4 percent PSA change subgroups. The median TTPP was 9.2 months for 30 %-decline, 6.5 months for non-decline, and 1.0 month for 25 %-elevation. In addition, of the 9 non-decline patients, 1 continued to have a PSA response and 6 showed PSA progression at EOT. Other 2 patients were censored by discontinuing of the treatment. Figure 2b shows TTPP according to the PSA response (≥50 %) at week 12. The median TTPP was 11.1 months for responders, 1.9 month for patients with PSA progression, and 6.5 months for others (non-response and non-progression).

**Table 2:** PSA kinetic parameters, mean ± SD (95 % CI)

	JPN-201 (*n* = 48)	JPN-202 (*n* = 46)
	Chemotherapy-naive	Chemotherapy-treated
Maximum % PSA decline	64.4 ± 38.3 (53.3–75.5)	19.7 ± 59.4 (2.0–37.3)
Time to PSA nadir (months)	5.3 ± 5.6 (3.7–6.9)	2.0 ± 3.4 (1.0–3.0)
Nadir PSA value (ng/mL)	19.5 ± 28.3 (11.3–27.7)	184.9 ± 282.3 (101.0–268.7)
PSA response (≥30 %) at week 12, n (%; 95 % CI)	35 (72.9; 58.2–84.7)	15 (32.6; 19.5–48.0)
PSA response (≥50 %) at week 12, n (%; 95 % CI)	29 (60.4; 45.3–74.2)	13 (28.3; 16.0–43.5)
PSA response (≥90 %) at week 12, n (%; 95 % CI)	9 (18.8; 8.9–32.6)	2 (4.3; 0.5–14.8)
PSA response (≥50 %) in treatment period, n (%; 95 % CI)	30 (62.5; 47.48–76.0)	13 (28.3; 16.0–43.5)
Time to PSA progression (months)	9.5 ± 7.4 (7.4–11.6)	3.8 ± 4.8 (2.4–5.2)

PSA prostate-specific antigen, *CI* confidence interval, *SD* standard deviation

**Fig. 3:**
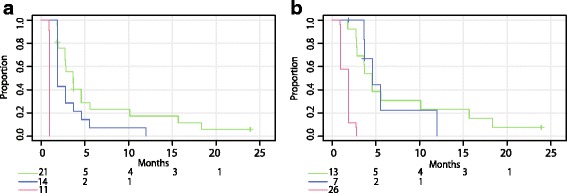
Kaplan–Meier curves for time to PSA progression in JPN-202. **a** green, PSA ≤ −30 % (30 %-PSA-decline); blue, −30 % < PSA < 25 % (non-decline); red, PSA ≥ 25 % (25 %-elevation) according to PSA change at week 4. **b** green, PSA response (≥50 %); blue, PSA non-response/progression; red, PSA progression according to PSA response at week 12

In JPN-202, the mean PSA decline at week 4 was 14.9 % (SD: 59.1) (Fig. 1c). Figure 1d depicts the percentage change of PSA transition for each patient. The PSA kinetic parameters are shown in Table 2. At week 4, 21 patients achieved a 30 %-decline, 14 patients showed a non-decline and 11 showed a 25 %-elevation. PSA responses (≥50 %) were confirmed in 13 of 21 30 %-decline patients, while none of the patients had PSA responses in the other subgroups. Figure 3a shows the TTPP according to the subgroups; the median TTPP was 3.7 months for 30 %-decline, 1.9 months for non-decline, and 1.0 month for 25 %-elevation. All non-30 %-decline patients had PSA progression at EOT. Figure 3b shows the TTPP according to PSA response (≥50 %) at week 12. The median TTPP was 4.6 months for responders, 1.9 months for patients with PSA progression, and 4.6 months for others (non-response and non-progression).

### Results of univariate Cox analyses

Table 3 shows the results of univariate Cox regression analyses for TTPP. In JPN-201, all variables showed significance except PSA decline (≥30 %) at week 4. In JPN-202, all variables showed significance except nadir PSA value and PSA response (≥90 %) at week 12.

**Table 3:** Results of univariate Cox analysis for time to PSA progression

	JPN-201		JPN-202	
	Chemotherapy-naive		Chemotherapy-treated	
	HR	95 % CI	HR	95 % CI
Maximum % PSA decline	0.975	0.964–0.986	0.981	0.975–0.987
Time to PSA nadir (months)	0.818	0.741–0.903	0.153	0.081–0.291
Nadir PSA value (ng/mL)	1.012	1.002–1.022	1.001	0.999–1.002
% PSA decline at week 4	0.989	0.981–0.998	0.980	0.973–0.986
% PSA decline at week 12	0.981	0.974–0.989	0.998	0.984–0.992
PSA decline (≥30 %) at week 4				
Non-decline (reference)	–	–	–	–
Decline	0.753	0.341–1.664	0.342	0.180–0.648
PSA response (≥30 %) at week 12				
Non-responder (reference)	–	–	–	–
Responder	0.331	0.158–0.692	0.335	0.167–0.674
PSA response (≥50 %) at week 12				
Non-responder (reference)	–	–	–	–
Responder	0.394	0.119–0.781	0.298	0.141–0.627
PSA response (≥90 %) at week 12				
Non-responder (reference)	–	–	–	–
Responder	0.357	0.137–0.930	0.807	0.193–3.369

*CI* confidence interval, *HR* hazard ratio, *PSA* prostate-specific antigen

## Discussion

In this post-hoc analysis, we first assessed the PSA kinetic profile in AA plus prednisolone-treated mCRPC patients. There was a clear difference in the PSA kinetic profile between chemotherapy-naïve and chemotherapy-treated patients. The majority of chemotherapy-naïve patients showed a rapid PSA decline at week 4, and additional 4 months were needed to reach PSA nadir. On the other hand, the majority of chemotherapy-treated patients showed non-decline or 25 % elevation at week 4, and the mean time to reach PSA nadir was 2.0 months. Moreover, the percent change in the PSA values in chemotherapy-treated patients tended to be larger, and most of the patients experienced PSA progression within a year after treatment initiation (Fig. 1). The mean TTPP was approximately 4 months, which was approximately half of the value reported in JPN-201 (9.5 months). Differences in characteristics of patient populations with mCRPC may impact the PSA kinetic profile, and such PSA kinetic differences could mainly be associated with previous history of chemotherapy. The mechanisms related to the differences in PSA kinetic profiles between chemotherapy-naïve and chemotherapy-treated patients are unclear; however, it is recommended that PSA kinetics would be separately evaluated according to prior history of chemotherapy. Caffo et al. reported that the time to PSA nadir in responders among chemotherapy-treated mCRPC patients was 15.5 weeks with AA but 7.0 weeks with enzalutamide [12]. Moreover, the anti-tumor effects of both drugs are derived from different mechanisms of action; abiraterone inhibits androgen synthesis at non-gonadal sites (adrenal gland and intratumorally) to reduce androgens to below castrate concentrations, while enzalutamide, an anti-androgen, directly binds androgen receptors to inhibit androgen receptor nuclear translocation [17]. These differences in the mechanisms of action might affect PSA kinetics.

In the present study, some patients showed a continuous stable level of PSA (Fig. 1b). In JPN-201, of the 9 (19 %) patients categorized as non-decline, most did not show large PSA elevation within 12 months. The TTPP curves were similar between non-decline and 30 %-decline patients (Fig. 2a). Thus, for chemotherapy-naïve patients without PSA progression at week 4, there would be a clinically similar chance of benefit from AA treatment. This is also supported by the result of Cox regression analysis, HR was significant for PSA response (≥50 %) at week 12 but not for PSA decline (≥30 %) at week 4 (Table 3).

On the other hand, in JPN-202, the non-decline TTPP curve was shorter than that of 30 %-decline and differed from that of 25 %-elevation (Fig. 3a). A half of the non-decline patients had rapid PSA progression; however, another half of the patients showed continuous stable levels of PSA (Fig. 1d), suggesting that patients with non-decline still had some potential of obtaining a clinical benefit from the treatment. The TTPP curves based on PSA response at week 12 were close between responders and patients with non-progression (Fig. 3b). Overall, further observation is recommended for patients in both population regardless obtaining PSA elevation at week 4.

In addition, temporal PSA elevation was recently reported with docetaxel treatment [18], which led to the phenomenon being referred to as “PSA flare,” indicating a PSA elevation with initiation of an LHRH agonist. PSA flares were also reported with AA treatment [19, 20]. According to the definition by Olbert et al., wherein a PSA flare is defined as initially rising PSA under therapy, dropping thereafter to values below baseline, 3 of 9 non-decline patients (Fig. 1b; blue lines) in JPN-201and 1 of 21 in JPN-202 (Fig. 1d; blue lines) were considered to have a PSA flare. PSA transition in such patients showed slow PSA elevations for 2 months, which declined below baseline levels within 1 to 2 months after the peak level. In taxane-based chemotherapy, the pattern of flare-up was slightly different. In the chemotherapy-related PSA flare, a peak PSA level was reached within 1 month after the first dose, and peak PSA levels were detected 1 to 2 months earlier than in the present study [18, 21, 22].

In general, elevated or stable PSA levels are a key decision factor for treatment change in routine clinical practice. Based on the aforementioned results, careful consideration is recommended for evaluation of the efficacy and decision for treatment change in AA treatment.

Some limitations of the present study are as follows: TTPP is a well-known surrogate endpoint for OS and is easy to follow-up in clinical practice [8]. In the present study, investigation of OS was inappropriate because patient number and number of events were limited. Therefore, we alternatively evaluated TTPP. Nonetheless, the primary endpoint in both JPN-201 and JPN-202 was PSA response rate at week 12; however, patient number was insufficient to apply statistical testing to analyze TTPP between the subgroups. Especially, patient number of non-30 %-decline was limited (11 in chemotherapy-naïve and 25 in chemotherapy-treated population). Moreover, in clinical practice, a wide variety of prior therapies, patient conditions, and concomitant use of drugs may have a potential impact on PSA kinetic profiles; it will therefore be important to reconfirm these results in clinical practice.

## Conclusions

Our results suggest that PSA kinetics and impact of early PSA decline on TTPP were not comparable and early PSA response showed slightly different association to TTPP according to prior history of chemotherapy. However, this is simply a descriptive study for which practice changing assessment of response or predictor of failure just cannot be made, further studies are warranted to confirm these results.

## Abbreviations

AA, abiraterone acetate; CI, confidence interval; ECOG-PS, Eastern Cooperative Oncology Group Performance Status; EOT, end of treatment; HR, hazard ratio; LHRH, luteinizing hormone-releasing hormone; mCRPC, metastatic castration-resistant prostate cancer; OS, overall survival; PCWG2, Prostate Cancer Clinical Trials Working Group; PSA, prostate-specific antigen; PSAWG, Prostate-Specific Antigen Working Group; SD, standard deviation; TTPP, time to prostate-specific antigen progression

## References

[1] Fitzmaurice C, Dicker D, Pain A, Hamavid H, Moradi-Lakeh M, MacIntyre MF, Allen C, Hansen G, Woodbrook R, Wolfe C (2015). The global burden of cancer 2013. JAMA Oncol.

[2] Huggins C, Masina MH, Eichelberger L, Wharton JD (1939). Quantitative studies of prostatic secretion : I. Characteristics of the normal secretion; the influence of thyroid, suprarenal, and tstis extirpation and androgen substitution on the prostatic output. J Exp Med.

[3] Barrie SE, Potter GA, Goddard PM, Haynes BP, Dowsett M, Jarman M (1994). Pharmacology of novel steroidal inhibitors of cytochrome P450(17) alpha (17 alpha-hydroxylase/C17-20 lyase). J Steroid Biochem Mol Biol.

[4] de Bono JS, Logothetis CJ, Molina A, Fizazi K, North S, Chu L, Chi KN, Jones RJ, Goodman OB, Saad F (2011). Abiraterone and increased survival in metastatic prostate cancer. N Engl J Med.

[5] Ryan CJ, Smith MR, de Bono JS, Molina A, Logothetis CJ, de Souza P, Fizazi K, Mainwaring P, Piulats JM, Ng S (2013). Abiraterone in metastatic prostate cancer without previous chemotherapy. N Engl J Med.

[6] Satoh T, Uemura H, Tanabe K, Nishiyama T, Terai A, Yokomizo A, Nakatani T, Imanaka K, Ozono S, Akaza H (2014). A phase 2 study of abiraterone acetate in Japanese men with metastatic castration-resistant prostate cancer who had received docetaxel-based chemotherapy. Jpn J Clin Oncol.

[7] Matsubara N, Uemura H, Satoh T, Suzuki H, Nishiyama T, Uemura H, Hashine K, Imanaka K, Ozono S, Akaza H (2014). A phase 2 trial of abiraterone acetate in Japanese men with metastatic castration-resistant prostate cancer and without prior chemotherapy (JPN-201 study). Jpn J Clin Oncol.

[8] Scher HI, Halabi S, Tannock I, Morris M, Sternberg CN, Carducci MA, Eisenberger MA, Higano C, Bubley GJ, Dreicer R (2008). Design and end points of clinical trials for patients with progressive prostate cancer and castrate levels of testosterone: recommendations of the Prostate Cancer Clinical Trials Working Group. J Clin Oncol.

[9] Armstrong AJ, Eisenberger MA, Halabi S, Oudard S, Nanus DM, Petrylak DP, Sartor AO, Scher HI (2012). Biomarkers in the management and treatment of men with metastatic castration-resistant prostate cancer. Eur Urol.

[10] Collette L, Burzykowski T, Carroll KJ, Newling D, Morris T, Schroder FH (2005). Is prostate-specific antigen a valid surrogate end point for survival in hormonally treated patients with metastatic prostate cancer? Joint research of the European Organisation for Research and Treatment of Cancer, the Limburgs Universitair Centrum, and AstraZeneca Pharmaceuticals. J Clin Oncol.

[11] Halabi S, Armstrong AJ, Sartor O, de Bono J, Kaplan E, Lin CY, Solomon NC, Small EJ (2013). Prostate-specific antigen changes as surrogate for overall survival in men with metastatic castration-resistant prostate cancer treated with second-line chemotherapy. J Clin Oncol.

[12] Caffo O, Veccia A, Maines F, Bonetta A, Spizzo G, Galligioni E (2014). Potential value of rapid prostate-specific antigen decline in identifying primary resistance to abiraterone acetate and enzalutamide. Future Oncol.

[13] Xu XS, Ryan CJ, Stuyckens K, Smith MR, Saad F, Griffin TW, Park YC, Yu MK, Vermeulen A, Poggesi I (2015). Correlation between prostate-specific antigen kinetics and overall survival in abiraterone acetate-treated castration-resistant prostate cancer patients. Clin Cancer Res.

[14] Guccione JR, Ledet EM, Stolten MD, Steinberger AE, Chow LD, Cotogno P, Lewis BE, Sartor AO (2016). Early assessment of PSA response in CRPC patients treated with enzalutamide (Enza) or abiraterone (Abi). ASCO Meeting Abstracts.

[15] Conteduca V, Crabb SJ, Scarpi E, Hanna C, Maines F, Joyce H, Fabbri P, Derosa L, Massari F, Lolli C (2016). Association between early PSA increase and clinical outcome in patients treated with enzalutamide for metastatic castration resistant prostate cancer. ASCO Meeting Abstracts.

[16] Bubley GJ, Carducci M, Dahut W, Dawson N, Daliani D, Eisenberger M, Figg WD, Freidlin B, Halabi S, Hudes G (1999). Eligibility and response guidelines for phase II clinical trials in androgen-independent prostate cancer: recommendations from the Prostate-Specific Antigen Working Group. J Clin Oncol.

[17] Hoffman-Censits J, Kelly WK (2013). Enzalutamide: a novel antiandrogen for patients with castrate-resistant prostate cancer. Clin Cancer Res.

[18] Olbert PJ, Hegele A, Kraeuter P, Heidenreich A, Hofmann R, Schrader AJ (2006). Clinical significance of a prostate-specific antigen flare phenomenon in patients with hormone-refractory prostate cancer receiving docetaxel. Anticancer Drugs.

[19] Burgio SL, Conteduca V, Rudnas B, Carrozza F, Campadelli E, Bianchi E, Fabbri P, Montanari M, Carretta E, Menna C (2015). PSA flare with abiraterone in patients with metastatic castration-resistant prostate cancer. Clin Genitourin Cancer.

[20] Narmala SK, Boulmay BC (2014). PSA flare after initiation of abiraterone acetate. J Community Support Oncol.

[21] Nelius T, Klatte T, de Riese W, Filleur S (2008). Impact of PSA flare-up in patients with hormone-refractory prostate cancer undergoing chemotherapy. Int Urol Nephrol.

[22] Angelergues A, Maillet D, Flechon A, Ozguroglu M, Mercier F, Guillot A, Le Moulec S, Gravis G, Beuzeboc P, Massard C (2014). Prostate-specific antigen flare induced by cabazitaxel-based chemotherapy in patients with metastatic castration-resistant prostate cancer. Eur J Cancer.

